# Greater low-density lipoprotein cholesterol variability is associated with increased progression to dialysis in patients with chronic kidney disease stage 3

**DOI:** 10.18632/oncotarget.23228

**Published:** 2017-12-14

**Authors:** Yu-Hsuan Lin, Jiun-Chi Huang, Pei-Yu Wu, Szu-Chia Chen, Yi-Wen Chiu, Jer-Ming Chang, Hung-Chun Chen

**Affiliations:** ^1^ School of Medicine, Kaohsiung Medical University, Kaohsiung, Taiwan; ^2^ Graduate Institute of Clinical Medicine, College of Medicine, Kaohsiung Medical University, Kaohsiung, Taiwan; ^3^ Division of Nephrology, Department of Internal Medicine, Kaohsiung Medical University Hospital, Kaohsiung Medical University, Kaohsiung, Taiwan; ^4^ Department of Internal Medicine, Kaohsiung Municipal Hsiao-Kang Hospital, Kaohsiung Medical University, Kaohsiung, Taiwan; ^5^ Department of Internal Medicine, Kaohsiung Municipal Cijin Hospital, Kaohsiung Medical University, Kaohsiung, Taiwan; ^6^ Faculty of Medicine, College of Medicine, Kaohsiung Medical University, Kaohsiung, Taiwan

**Keywords:** LDL-cholesterol variability, chronic kidney disease, dialysis

## Abstract

Increasing evidence suggests that lipid variability may be a predictor of cardiovascular events. However, few studies have evaluated the association between lipid variability and renal outcomes in patients with moderate-to-advanced chronic kidney disease (CKD). Therefore, the aims of this study were to assess whether lipid variability is associated with progression to dialysis in patients with CKD stage 3–5, and to evaluate the risk factors of lipid variability. This longitudinal study enrolled 725 patients with CKD stage 3–5. Intra-individual lipid variability was defined as the standard deviations (SDs) of lipid levels. The renal end-point was defined as commencing dialysis. During a mean follow-up period of 3.2 years, 208 patients (28.7%) started dialysis. The patients with CKD stage 3 with high low-density lipoprotein (LDL) cholesterol SD (per 1 mg/dL; hazard ratio, 1.035; 95% confidence interval, 1.003 to 1.067; *p* = 0.003) were associated with an increased risk of progression to dialysis, however this association was not seen in the patients with CKD stage 4 or 5. Furthermore, in the patients with CKD stage 3, a high urine protein-to-creatinine ratio (*p* < 0.001) and the use of statins (*p* < 0.001) were significantly associated with an increased LDL-cholesterol SD. Greater LDL-cholesterol variability was associated with an increased risk of progression to dialysis in patients with CKD stage 3, but not in those with CKD stage 4 or 5. These findings support the potential role of aggressive lipid control on clinical outcomes and highlight its importance in patients with CKD stage 3.

## INTRODUCTION

Chronic kidney disease (CKD) severely impairs key enzymes and metabolic pathways, ultimately leading to the dysregulation of high-density lipoprotein (HDL) cholesterol and triglyceride-rich lipoproteins [1]. Previous studies on lipid profiles have reported hypertriglyceridemia, hypercholesterolemia, higher levels of low-density lipoprotein (LDL) cholesterol, and lower levels of HDL-cholesterol in patients with CKD [2, 3]. The progression of CKD can worsen these metabolic derangements, potentially leading to atherogenic diathesis and a further decline in renal function^2^. Experimental studies have suggested that hyperlipidemia worsens renal damage through tubulointerstitial disease and progressive glomerulosclerosis [4, 5]. In addition, increasing evidence suggests that abnormal lipid metabolism can promote the progression of renal disease [6–8]. Clinical studies on the association between the use of statins and progression of renal disease in patients with mild-to-moderate kidney failure have reported inconsistent findings, although some have suggested that statin therapy can decrease the rate of decline in renal function [9–11].

Several epidemiologic studies have reported that dyslipidemia is associated with cardiovascular morbidity and mortality in the general population [12, 13]. In recent years, lipid variability has been reported to be a reliable predictor of cardiovascular events [14–18]. Lipid variability can cause instability of the vascular wall due to variability in the lipid efflux mechanism. This can then increase vulnerability to plaque rupture, and increase the risk of cardiovascular events. With regards to renal outcomes, Ceriello [19] and Chang [20] found that lipid variability can predict the development of diabetic nephropathy in diabetic patients with preserved renal function. However, published data regarding the relationship between lipid variability and renal outcomes in patients with moderate-to-advanced CKD are limited. Therefore, the aims of this study were to assess whether lipid variability is associated with progression to dialysis in patients with CKD stage 3–5, and to evaluate the risk factors of lipid variability.

## RESULTS

A total of 725 patients with CKD stage 3–5 were included. Their mean age was 64.4 ± 12.1 years, and there were 460 males and 265 females. All of the patients were divided into two groups according to whether or not they progressed to dialysis. The clinical characteristics are shown in Table 1. Compared to patients who did not progress to dialysis, those who did progress to dialysis were younger, had a higher prevalence of coronary artery disease, higher urine protein-to-creatinine ratio (U_PCR_), more advanced CKD stage, lower levels of albumin, baseline estimated glomerular filtration rate (eGFR), hemoglobin, and total calcium, higher levels of phosphorous and uric acid, and higher standard deviation (SD) levels of triglycerides, total cholesterol, HDL-cholesterol and LDL-cholesterol. In addition, the patients who progressed to dialysis used more angiotensin converting enzyme inhibitors (ACEIs), angiotensin II receptor blockers (ARBs), calcium channel blockers and diuretics.

**Table 1:** Comparison of clinical characteristics according to patients without or with progression to dialysis in all study patients

Characteristics	Without progression to dialysis (*n* = 517)	With progression to dialysis (*n* = 208)	*p*	All (*n* = 725)
Age (year)	65.9 ± 11.7	60.7 ± 12.1	<0.001	64.4 ± 12.1
Male gender (%)	65.5	58.7	0.083	63.5
Smoking history (%)	31.5	26.9	0.216	30.2
Diabetes mellitus (%)	60.5	67.1	0.097	62.4
Hypertension (%)	87.4	89.9	0.357	88.1
Coronary artery disease (%)	11.1	18.4	0.009	13.2
Cerebrovascular disease (%)	12.4	8.2	0.106	11.2
CKD stage				
Stage 3 (%)	48.5	9.1	<0.001	37.2
Stage 4 (%)	36.9	31.7		35.4
Stage 5 (%)	14.5	59.1		27.3
CKD etiology			0.001	
Glomerulonephritis (%)	21.5	23.1		21.9
Diabetic nephropathy (%)	56.9	65.4		59.3
Obstructive nephropathy (%)	4.3	1.4		3.4
Gouty nephropathy (%)	7.4	1.0		5.5
Polycystic kidney disease (%)	1.5	4.3		2.3
Malignant hypertension (%)	6.6	3.8		5.8
Others (%)	1.9	1.0		1.7
Laboratory parameters				
Albumin (g/dL)	4.1 ± 0.4	3.8 ± 0.5	<0.001	4.0 ± 0.4
Fasting glucose (mg/dL)	128.4 ± 53.9	126.4 ± 60.6	0.678	127.8 ± 55.8
Hemoglobin (g/dL)	11.9 ± 2.2	10.1 ± 1.8	<0.001	11.4 ± 2.2
Baseline eGFR (mL/min/1.73 m^2^)	29.7 ± 12.4	15.6 ± 9.6	<0.001	25.6 ± 13.3
Total calcium (mg/dL)	9.4 ± 0.7	8.9 ± 1.1	<0.001	9.3 ± 0.8
Phosphorous (mg/dL)	3.8 ± 0.7	5.0 ± 1.5	<0.001	4.1 ± 1.2
Uric acid (mg/dL)	8.1 ± 2.1	8.8 ± 2.0	<0.001	8.3 ± 2.1
Mean triglyceride (mg/dL)	169.2 ± 119.4	171.7 ± 90.6	0.759	169.9 ± 110.9
SD triglyceride (mg/dL)	59.0 ± 59.5	71.7 ± 64.4	0.011	62.6 ± 61.2
Mean total cholesterol (mg/dL)	188.7 ± 32.0	190.0 ± 35.8	0.629	189.1 ± 33.1
SD total cholesterol (mg/dL)	31.2 ± 16.8	37.1 ± 21.0	<0.001	32.9 ± 18.3
Mean HDL-cholesterol (mg/dL)	43.8 ± 11.1	43.8 ± 16.7	0.987	43.8 ± 12.9
SD HDL-cholesterol (mg/dL)	6.0 ± 3.8	7.3 ± 3.6	<0.001	6.4 ± 3.8
Mean LDL-cholesterol (mg/dL)	107.4 ± 55.1	102.5 ± 23.9	0.221	106.0 ± 48.3
SD LDL-cholesterol (mg/dL)	24.4 ± 13.0	28.0 ± 16.4	0.005	25.4 ± 14.1
U_PCR_ (mg/g)	1681.5 ± 2385.8	4075.7 ± 3631.1	<0.001	2441.4 ± 3047.2
Medications				
ACEI and/or ARB use (%)	65.0	48.6	<0.001	60.3
β-blocker use (%)	19.1	24.0	0.141	20.6
Calcium channel blocker use (%)	35.2	55.2	<0.001	41.0
Diuretics use (%)	10.4	22.1	<0.001	13.8
Statin use (%)	32.1	29.3	0.465	31.3

Abbreviations. CKD, chronic kidney disease; eGFR, estimated glomerular filtration rate; SD, standard deviation; HDL, high-density lipoprotein; LDL, low-density lipoprotein; ACEI, angiotensin converting enzyme inhibitor; ARB, angiotensin II receptor blocker.

### Risk factors for progression to dialysis among different stages of CKD

The median follow-up period was 3.2 (1.2–6.3) years. During the follow-up period, 208 patients developed end-stage renal disease, of whom 197 started hemodialysis and 11 started peritoneal dialysis.

**Figure 1:**
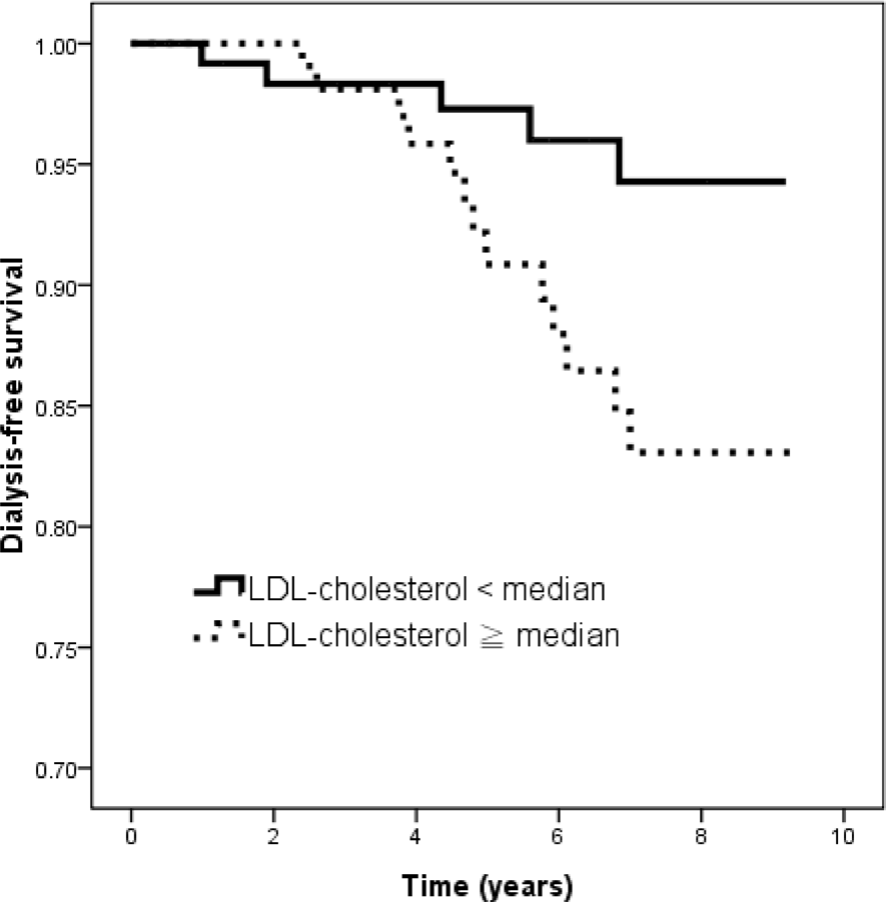
Kaplan–Meier analysis of dialysis-free survival according to median of LDL-cholesterol SD (log-rank *p* = 0.005) in CKD stage 3 patients Patients with LDL-cholesterol SD < median had a better renal-free survival than those with LDL-cholesterol SD ≥ median.

In CKD stage 3 patients, compared to patients who did not progress to dialysis, those who did progress to dialysis were younger, lower albumin, lower baseline eGFR, higher total calcium, higher phosphorous, higher SD HDL-cholesterol and higher SD LDL-cholesterol. Table 2 lists the hazard ratios (HRs) of the risk factors for progression to dialysis using Cox proportional hazards analysis among CKD stage 3 patients. After adjustments for age, albumin, baseline eGFR, total calcium, phosphorous, SD HDL-cholesterol and SD LDL-cholesterol, the patents with CKD stage 3 with high phosphorous (per 1 mg/dL; HR, 4.720; 95% confidence interval [CI], 1.664 to 13.384; *p* = 0.004) and high LDL-cholesterol SD (per 1 mg/dL; HR, 1.035; 95% CI, 1.003 to 1.067; *p* = 0.003) were associated with an increased risk of progression to dialysis in the adjusted model. We have further performed correlation matrix of regression coefficients, and the correlation coefficients is -0.065 between phosphorous and SD LDL-cholesterol. Figure 1 illustrates the Kaplan-Meier curves for dialysis-free survival (log-rank *p* = 0.035) for the patients with CKD stage 3 subdivided according to the median LDL-cholesterol SD. The patients with a LDL-cholesterol SD less than the median value had a better renal-free survival than those with a LDL-cholesterol SD equal to or higher than the median value.

**Table 2:** Risk factors for progression to dialysis using multivariate forward cox proportional hazards model among CKD stage 3

Parameters	Multivariate (forward)	
	Hazard ratios (95% CI)	*p*
Phosphorous (per 1 mg/dL)	4.720 (1.664–13.384)	0.004
SD LDL-cholesterol (per 1 mg/dL)	1.035 (1.003–1.067)	0.003

Values expressed as Hazard Ratios and 95% confidence interval (CI). Abbreviations are same as Table 1.

Adjusted for age, albumin, baseline eGFR, total calcium, phosphorous, SD HDL-cholesterol and SD LDL-cholesterol.

For CKD stage 4, compared to patients who did not progress to dialysis, those who did progress to dialysis were younger, male predominant, diabetes, lower albumin, lower hemoglobin, lower baseline eGFR, lower total calcium, higher phosphorous, higher SD LDL-cholesterol, higher UPCR, and higher percentage of calcium channel blockers and diuretics use. In Table 3, After adjustments for age, gender, diabetes mellitus, albumin, baseline eGFR, hemoglobin, total calcium, phosphorous, SD LDL-cholesterol, U_PCR_, calcium channel blocker use and diuretic use, CKD stage 4 patients who were younger (per 1 year; HR, 0.953; 95% CI, 0.932 to 0.975; *p* < 0.001), with a history of diabetes (HR, 4.698; 95% CI, 2.237 to 9.865; *p* < 0.001), low albumin (per 1 g/dL; HR, 0.281; 95% CI, 0.148 to 0.533; *p* < 0.001), low baseline eGFR (per 1 mL/min/1.73 m^2^; HR, 0.891; 95% CI, 0.839 to 0.947; *p* < 0.001), low total calcium (per 1 mg/dL; HR, 0.581; 95% CI, 0.371 to 0.909; *p* = 0.017), used calcium channel blockers (HR, 2.283; 95% CI, 1.334 to 3.909; *p* = 0.003) and used diuretics (HR, 1.960; 95% CI, 1.008 to 3.813; *p* = 0.047) were associated with an increased risk of dialysis in the adjusted model. We have further performed correlation matrix of regression coefficients, and the correlation coefficients are all <0.7.

**Table 3:** Risk factors for progression to dialysis using multivariate forward cox proportional hazards model among CKD stage 4

Parameters	Multivariate (forward)	
	Hazard ratios (95% CI)	*p*
Age (per 1 year)	0.953 (0.932–0.975)	<0.001
Diabetes mellitus	4.698 (2.237–9.865)	<0.001
Albumin (per 1 g/dL)	0.281 (0.148–0.533)	<0.001
Baseline eGFR (per 1 mL/min/1.73 m^2^)	0.891 (0.839–0.947)	<0.001
Total calcium (per 1 mg/dL)	0.581 (0.371–0.909)	0.017
Calcium channel blocker use	2.283 (1.334–3.909)	0.003
Diuretics use	1.960 (1.008–3.813)	0.047

Values expressed as Hazard Ratios and 95% confidence interval (CI). Abbreviations are same as Table 1.

Adjusted for age, gender, diabetes mellitus, albumin, baseline eGFR, hemoglobin, total calcium, phosphorous, SD LDL-cholesterol, U_PCR_, calcium channel blocker use and diuretic use.

In addition, for CKD stage 5, compared to patients who did not progress to dialysis, those who did progress to dialysis were younger, lower baseline eGFR, lower total calcium, higher phosphorous and higher U_PCR_. After adjustments for age, baseline eGFR, total calcium, phosphorous and U_PCR_ (Table 4), CKD stage 5 patients with low baseline eGFR (per 1 mL/min/1.73 m^2^; HR, 0.904; 95% CI, 0.839 to 0.974; *p* = 0.008), high phosphorous (per 1 mg/dL; HR, 1.483; 95% CI, 1.280 to 1.719; *p* < 0.001) and high U_PCR_ (per 1 mg/g; HR, 5.489; 95% CI, 3.090 to 9.752; *p* < 0.001) were associated with an increased risk of dialysis in the adjusted model. We have further performed correlation matrix of regression coefficients, and the correlation coefficients are all <0.7.

**Table 4:** Risk factors for progression to dialysis using multivariate forward cox proportional hazards model among CKD stage 5

Parameters	Multivariate (forward)	
	Hazard ratios (95% CI)	*p*
Baseline eGFR (per 1 mL/min/1.73 m^2^)	0.904 (0.839–0.974)	0.008
Phosphorous (per 1 mg/dL)	1.483 (1.280–1.719)	<0.001
U_PCR_ (per 1 mg/g)	5.489 (3.090–9.752)	<0.001

Values expressed as Hazard Ratios and 95% confidence interval (CI). Abbreviations are same as Table 1.

Adjusted for age, baseline eGFR, total calcium, phosphorous and U_PCR_.

### LDL-cholesterol change and the correlation between LDL- and HDL-cholesterol over the follow-up period

**Figure 2:**
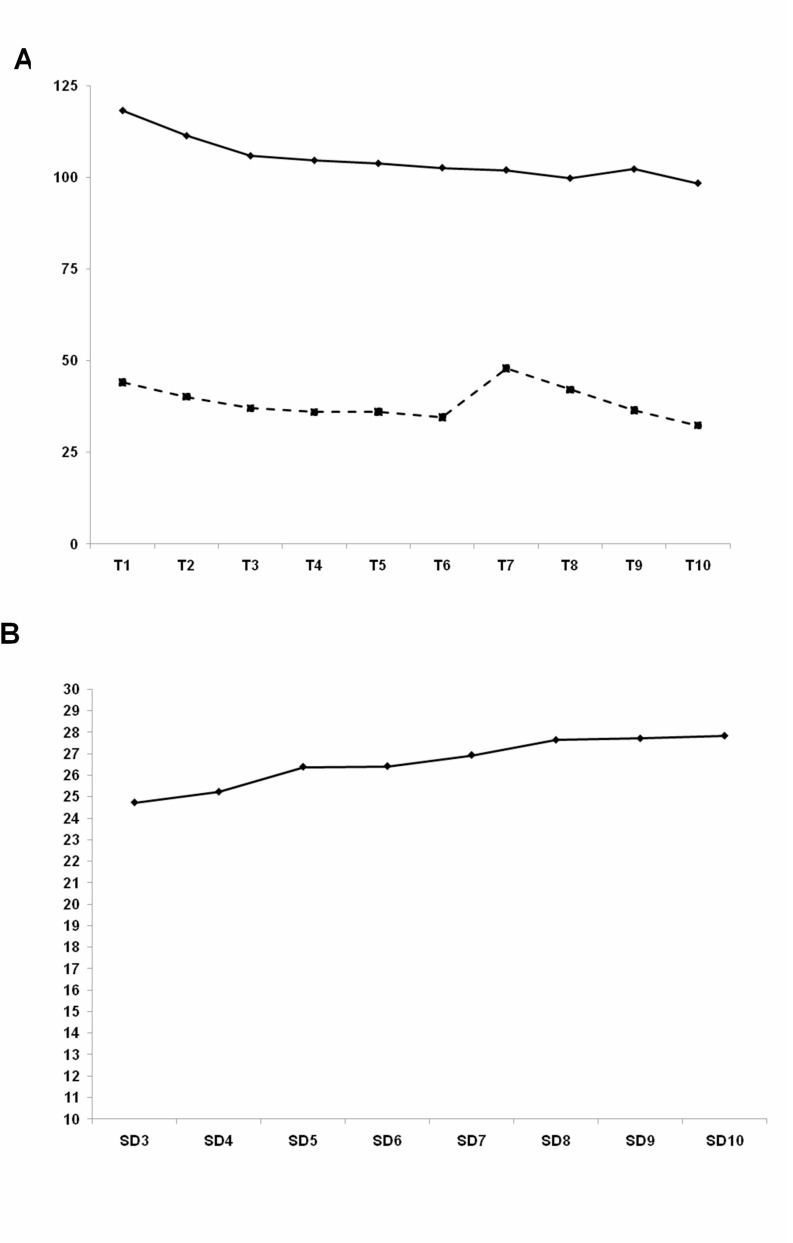
(**A**) LDL-cholesterol (solid line) and SD LDL-cholesterol (dashed line) change through follow-up period. (**B**) Values of SD3 (SD of 1st to 3rd LDL-cholesterol) to SD10 (SD of 1st to 10th LDL-cholesterol).

**Figure 3:**
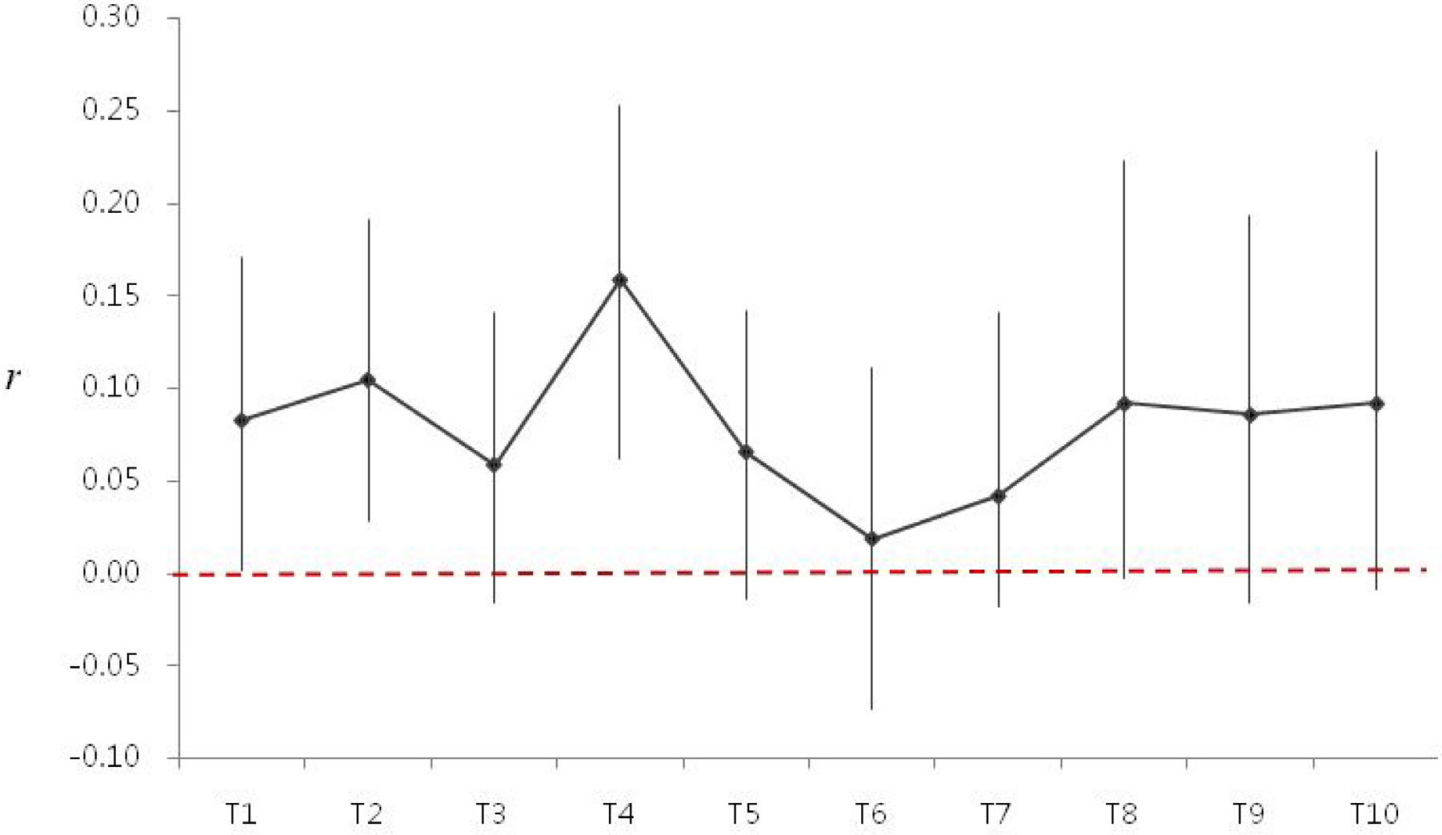
The association between LDL- and HDL-cholesterol was expressed using Pearson’s *r* expression

Figure 2A illustrates LDL-cholesterol (solid line) and SD LDL-cholesterol (dashed line) change through follow-up period, and Figure 2B shows the values of SD3 (SD of 1st to 3rd LDL-cholesterol) to SD10 (SD of 1st to 10th LDL-cholesterol). Besides, Figure 3 demonstrates the relation between LDL- and HDL-cholesterol using Pearson’s *r*.

### Determinants of LDL-cholesterol variability in the patients with CKD stage 3

Comparisons of the clinical characteristics between the patients with CKD stage 3 with LDL-cholesterol SD < the median value and ≥ the median value are shown in Table 5. Compared to the patients with LDL-cholesterol SD < the median value, those with LDL-cholesterol SD ≥ the median value had a higher prevalence of diabetes mellitus (DM), higher fasting glucose level, higher U_PCR_, and higher percentage of statin use.

**Table 5:** Comparison of clinical characteristics according to LDL-cholesterol SD < median or ≥ median in CKD stage 3 patients

Characteristics	LDL-cholesterol SD < median (*n* = 135)	LDL-cholesterol SD ≥ median (*n* = 135)	*p*	All (*n* = 270)
Age (year)	65.6 ± 11.8	62.8 ± 11.7	0.051	64.2 ± 11.8
Male gender (%)	76.3	76.3	1.000	76.3
Smoking history (%)	34.3	36.3	0.736	35.3
Diabetes mellitus (%)	48.9	68.1	0.001	58.5
Hypertension (%)	88.1	88.1	0.982	88.1
Coronary artery disease (%)	8.2	7.5	0.834	7.9
Cerebrovascular disease (%)	9.6	3.4	0.256	7.8
Laboratory parameters				
Albumin (g/dL)	4.2 ± 0.2	4.1 ± 0.4	0.112	4.2 ± 0.3
Fasting glucose (mg/dL)	116.8 ± 37.1	140.7 ± 65.8	<0.001	128.8 ± 54.7
Hemoglobin (g/dL)	12.9 ± 1.9	13.0 ± 1.9	0.718	13.0 ± 1.9
Baseline eGFR (mL/min/1.73 m^2^)	40.1 ± 7.0	39.9 ± 7.0	0.764	40.0 ± 6.9
Total calcium (mg/dL)	9.6 ± 0.5	9.5 ± 0.6	0.678	9.6 ± 0.6
Phosphorous (mg/dL)	3.5 ± 0.5	3.6 ± 0.5	0.769	3.6 ± 0.5
Uric acid (mg/dL)	7.7 ± 1.9	7.9 ± 2.0	0.413	7.8 ± 1.9
U_PCR_ (mg/g)	748.6 ± 1079.2	1535.9 ± 2398.4	0.004	1144.3 ± 1899.5
Medications				
ACEI and/or ARB use (%)	71.9	72.6	0.892	72.2
β-blocker use (%)	17.0	14.8	0.618	15.9
Calcium channel blocker use (%)	35.6	28.1	0.191	31.9
Diuretics use (%)	5.2	7.4	0.452	6.3
Statin use (%)	48.9	14.8	<0.001	31.9

Abbreviations are same as Table 1.

Table 6 shows the determinants of LDL-cholesterol SD in the patients CKD with stage 3. In the univariate analysis, LDL-cholesterol SD was significantly positively correlated with DM, hypertension, fasting glucose, phosphorous, U_PCR_, and the use of statins, and negatively correlated with albumin. In the multivariate stepwise analysis, high U_PCR_ (unstandardized coefficient β, 0.003; 95% CI, 0.002 to 0.004; *p* < 0.001) and the use of statins (unstandardized coefficient β, 12.199; 95% CI, 9.173 to 15.224; *p* < 0.001) were significantly associated with an increased LDL-cholesterol SD.

**Table 6:** Determinants of LDL-cholesterol SD in CKD stage 3 patients using linear regression analysis

Parameter	Univariate		Multivariate (stepwise)	
	Unstandardized coefficient β (95% CI)	*p*	Unstandardized coefficient β (95% CI)	*p*
Age (per 1 year)	–0.103 (–0.227, 0.022)	0.105	–	–
Male (*vs.* female)	0.708 (–2.767, 4.184)	0.689	–	–
Smoking history	0.468 (–2.632, 3.569)	0.766	–	–
Diabetes mellitus	4.899 (1.957, 7.841)	0.001	–	–
Hypertension	2.963 (–1.592, 7.518)	0.201	–	–
Coronary artery disease	–1.814 (–7.345, 3.717)	0.519	–	–
Cerebrovascular disease	–2.351 (–7.864, 3.162)	0.402	–	–
Laboratory parameters				
Albumin (per 1 g/dL)	–8.394 (–12.575, –4.213)	<0.001	–	–
Fasting glucose (per 1 mg/dL)	0.048 (0.021, 0.075)	0.001	–	–
Hemoglobin (per 1 g/dL)	–0.047 (–0.838, 0.743)	0.906	–	–
Baseline eGFR (per 1 mL/min/1.73 m^2^)	–0.102 (–0.315, 0.111)	0.346	–	–
Total calcium (per 1 mg/dL)	–1.214 (–3.774, 1.346)	0.351	–	–
Phosphorous (per 1 mg/dL)	1.570 (–1.408, 4.549)	0.300	–	–
Uric acid (per 1 mg/dL)	0.160 (–0.609, 0.929)	0.682	–	–
U_PCR_ (per 1 mg/g)	0.003 (0.002, 0.003)	<0.001	0.003 (0.002, 0.004)	<0.001
Medications				
ACEI and/or ARB use	0.803 (–2.497, 4.102)	0.632	–	–
β-blocker use	0.042 (–3.998, 4.082)	0.984	–	–
Calcium channel blocker use	–0.702 (–3.874, 2.470)	0.663	–	–
Diuretics use	4.460 (–1.603, 10.523)	0.149	–	–
Statin use	11.008 (8.124, 13.892)	<0.001	12.199 (9.173, 15.224)	<0.001

Values expressed as unstandardized coefficient β and 95% confidence interval (CI). Abbreviations are same as Table 1**.**

### LDL-cholesterol values in different CKD etiologies

We have further performed Table 7 to show the values of mean and SD LDL-cholesterol among groups of different CKD etiologies, and find patient with DM had higher values of SD LDL-cholesterol than glomerulonephritis, gouty nephropathy, polycystic kidney disease and malignant hypertension.

**Table 7:** Values of mean and SD LDL-cholesterol among groups of different CKD etiologies

Parameters	Glomerulonephritis (*n* = 159)	Diabetic nephropathy (*n* = 430)	Obstructive nephropathy (*n* = 25)	Gouty nephropathy (*n* = 40)	Polycystic kidney disease (*n* = 17)	Malignant hypertension (*n* = 42)	Others (*n* = 12)	*p*
Mean LDL-cholesterol (mg/dL)	108.1 ± 26.3	106.5 ± 58.6	103.2 ± 35.4	100.9 ± 25.8	91.4 ± 29.3	104.0 ± 21.8	111.0 ± 27.4	0.854
SD LDL-cholesterol (mg/dL)	22.3 ± 12.6^*^	28.1 ± 14.8	24.0 ± 13.3	19.5 ± 10.6^*^	17.3 ± 10.0^*^	19.5 ± 11.5^*^	26.3 ± 8.5	<0.001

Values expressed as mean ± SD.

^*^p < 0.05 compared to diabetic nephropathy.

### Relation of SD LDL-cholesterol with double creatinine or dialysis among different stages of CKD

The median follow-up period was 2.8 (1.0–5.7) years. During the follow-up period, 312 patients (43.0%) developed double creatinine or dialysis. In CKD stage 3 patients, high LDL-cholesterol SD (per 1 mg/dL; HR, 1.037; 95% CI, 1.015 to 1.060; *p* = 0.001) were associated with an increased risk for double creatinine or dialysis in univariate analysis, but not achieving significance (*p* = 0.059) in the multivariate analysis. Similarly, in CKD stage 4 patients, high LDL-cholesterol SD (per 1 mg/dL; HR, 1.011; 95% CI, 1.001 to 1.020; *p* = 0.036) was associated with double creatinine or dialysis in univariate analysis, but not achieving significance (*p* = 0.065) in the multivariate analysis. However, in CKD stage 5 patients, LDL-cholesterol SD (per 1 mg/dL; HR, 1.003; 95% CI, 0.992 to 1.004; *p* = 0.557) was not associated with double creatinine or dialysis in univariate analysis.

## DISCUSSION

The present study evaluated the association between lipid variability and renal outcomes in patients with CKD stage 3–5 over a follow-up period of 3.2 years. The results showed that the patients with CKD stage 3 with increased LDL-cholesterol SD had an increased risk of progression to dialysis. However, this relationship was not significant for those with CKD stage 4 or 5. Furthermore, a high U_PCR_ and the use of statins were associated with an increased LDL-cholesterol SD in the patients with CKD stage 3.

The most important finding of the present study is that greater LDL-cholesterol variability was associated with a higher risk of progression to dialysis in the patients with CKD stage 3, which suggests that a more stable and less variable LDL-cholesterol level is important. It seems that maintaining certain biological parameters within a very narrow range is crucial. For example, glycemia should always be maintained in healthy people between 3.89 and 7.78 mmol/L [21]. Strictly maintaining glucose values within this range has been strongly associated with increased survival in non-diabetic, critically ill adults [22]. Similarly, blood pressure variability has been identified as a cardiovascular risk factor in people without diabetes [23]. Ceriello [19] evaluated the effect of lipid variability on renal outcomes in patients with type 2 DM with preserved renal function, and found that higher a HDL-cholesterol level resulted in a higher risk of developing albuminuria, and that HDL- and LDL-cholesterol variability predicted a decline in eGFR of <60 mL/min/1.73 m^2^. The pathophysiological link between dyslipidemia and CKD has been reported to be due to worsening atherosclerosis in the renal microcirculation and accumulation of lipoprotein in glomerular structures. This then stimulates the release of cytokines and growth factors, subsequently leading to inflammation and fibrogenesis [24, 25]. Animal studies have shown that higher levels of total cholesterol can increase the rate of progression of kidney disease, and that a diet high in cholesterol can cause macrophage infiltration and the formation of foam cells [24, 26]. In the present study, the impact of LDL-cholesterol variability on the risk of progression to dialysis in the patients with CKD stage 3 remained significant after adjustments for mean LDL-cholesterol. This suggests that LDL-cholesterol variability may provide additional valuable information as a potential predictor of adverse renal outcomes.

The second important finding of this study is that the significant association between lipid variability and progression to dialysis was not observed in the patients with CKD stage 4 or 5. This implies that the predictive power of lipid variability on the risk of adverse renal outcomes among patients with CKD stage 4 or 5 may be relatively low. The prognostic role of lipid variability in such patients is unclear, but it may be due to a paradoxical relationship of a high prevalence of malnutrition and inflammation [27, 28]. The concept of reverse epidemiology has recently been proposed, which questions the applicability of traditional cardiovascular risk factors and the necessity of pharmaceutical management in patients with renal failure [24, 25]. Rather, the concept of reverse epidemiology proposes that malnutrition and inflammation are more important than traditional factors. Thus, although aggressive lipid control may be beneficial in slowing the progression of renal disease in patients with mild-to-moderate kidney failure [9–11], further studies are needed to investigate whether tight lipid control can improve the renal outcomes of patients with advanced stages of CKD.

Another important finding of this study is that proteinuria was associated with LDL-cholesterol variability in the patients with CKD stage 3. Proteinuria is a common finding in patients with CKD, irrespective of the cause, and almost all patients with CKD present with varying degrees of proteinuria [29]. Proteinuria is therefore important as a marker of renal disease, and it is also associated with catabolic processes, protein-energy wasting, hypoalbuminemia, and inflammation [30, 31]. Furthermore, in patients with CKD, proteinuria may contribute to dysregulated lipoprotein catabolism and increased oxidative stress [32]. Proteinuria has been associated with increased transcapillary escape rates of albumin in patients with diabetes and essential hypertension [33], and it has also been suggested that this could reflect a generalized vasculopathy secondary to endothelial damage [34]. This could result in disordered hemostasis and progressive atherosclerosis if endothelial fibrinolytic activity is compromised and if the loss of endothelial integrity also leads to enhanced transvascular escape of atherogenic macromolecules such as modified LDL-cholesterol. Taken together, our findings suggest that proteinuria level is a main factor influencing lipid variability in patients with moderate CKD.

The present study shows that patients with DM had greater LDL-cholesterol variability than patients with other etiologies of CKD. Many lipoprotein abnormalities are seen in the untreated, hyperglycemic diabetic patient due to overproduction of TG-rich lipoproteins in the liver. Diabetic patients may have mild hypertriglyceridemia, increased intermediate-density lipoprotein levels, small dense LDL with increased apoprotein B, and decreased HDL-cholesterol levels. The central, abdominal distribution of adipose tissue is associated with insulin resistance, hypertension, and the above lipoprotein abnormalities [35]. In our study, compared to non-diabetic patients, diabetic patients had higher prevalence of statins use (39.4% *vs.* 18.0%, *p* < 0.001), which may explain the high LDL-cholesterol SD in diabetic patients in our study. Biologically, chronic statin therapy may have modified LDL-cholesterol receptors, reduced LDL-cholesterol variability and led to better clinical outcomes. However, previous study demonstrated that LDL-cholesterol variability was associated with compliance of statin [36]. Therefore, improving adherence plays an important role to lower the variability and to stabilize LDL-cholesterol. Moreover, greater LDL- and HDL-cholesterol variability is associated with decline in eGFR and progression to albuminuria, respectively, in diabetic patients [19] , suggesting that more consistent and less variable values are desirable.

There are several limitations to this study. First, as an observational study, the number and frequency of lipid measurements varied between individual patients. To minimize this effect on the results, patients with fewer than three lipid measurements during the follow-up period and those with a follow-up period shorter than 6 months were excluded. However, the lack of uniformity of such measurements remains an important limitation of the analysis. Moreover, information on the use of statins was collected at the beginning of the study, however data about the duration, dosage and adherence to the medications were lacking. Therefore, we were unable to evaluate the influence of statins on lipid variability and/or renal outcomes. Further studies are needed to determine whether lipid-lowering agents are helpful in improving lipid variability. Lastly, the effect of anti-hypertensive medications on renal outcomes was not evaluated because this study was not a clinical trial aimed at investigating the effects of medications. We also lacked sufficient data on cumulative exposure duration and defined daily dose, and the positive correlation between the use of anti-hypertensive medications and renal outcomes may be due to selection bias.

In conclusion, greater LDL-cholesterol variability was associated with an increased risk of progression to dialysis among patients with CKD stage 3, but not in patients with CKD stage 4 or 5. These findings support the potential role of aggressive lipid control to improve clinical outcomes, and highlight its importance in patients with CKD stage 3.

## MATERIALS AND METHODS

### Patients and study design

This study was performed at a hospital in southern Taiwan, and included patients with CKD stages 3 to 5 defined as an eGFR (mL/min/1.73 m^2^) of 30 to 59, 15 to 29, and <15, respectively, for 3 months or longer [37] from January 2007 to September 2015. All of the included patients were regularly followed-up at our outpatient clinics. The exclusion criteria were patients with fewer than three lipid measurements during the follow-up period, and those who died or started dialysis therapy within 6 months after enrollment to avoid incomplete observations of changes in renal function. In total, 725 patients (mean age 64.4 ± 12.1 years, 460 males) were included in this study. The study protocol was approved by the Institutional Review Board of our hospital, and all of the enrolled patients provided written, informed consent.

### Collection of demographic, medical, and laboratory data

Demographic and medical data including age, gender and details of any comorbidities were recorded from the patients’ medical charts or in interviews. Laboratory tests were performed on fasting blood samples using a COBAS Integra 400 autoanalyzer (Roche Diagnostics GmbH, Mannheim, Germany), including levels of serum creatinine which were assessed using the compensated Jaffé method [38]. eGFR was calculated according to the method proposed in the Modification of Diet in Renal Disease study [39]. Blood samples were obtained within 1 month of enrollment. In addition, data regarding the use of ACEIs, ARBs, β-blockers, calcium channel blockers, diuretics, and statins during the study period were obtained from the patients’ medical records.

### Serial lipid measurements

Lipid measurements including triglycerides, total cholesterol, HDL-cholesterol and LDL-cholesterol were recorded for all patients from the date of enrollment until the development of the renal end-point or April 2016, whichever occurred first. Intrapersonal means and SDs of lipid levels were calculated for each patient, and the SD was considered to be an index of lipid variability.

### Definition of renal endpoint

The renal endpoint was defined as initiating dialysis. In Taiwan, dialysis is started according to the National Health Insurance program regulations, which are based on laboratory data, nutritional status and uremia. Data on renal function were censored in the patients who reached the renal endpoint, while the remaining patients were followed until April 2016.

Another renal endpoint was defined as double creatinine level or dialysis (which came first) since the enrollment of the patients. For patients who were reaching double creatinine or dialysis, the renal function data were censored. The patients who did not reach renal endpoints were followed up until April 2016.

### Statistical analysis

Statistical analysis was performed using SPSS version 19.0 for Windows (SPSS Inc. Chicago, USA). Data are expressed as percentages or means ± SDs. Differences between groups were analyzed using the chi-square test for categorical variables and the independent *t*-test for continuous variables. The time to the renal endpoint and covariates of risk factors were modeled using a Cox proportional hazards model. Survival curves for the renal endpoint were obtained using the Kaplan-Meier method. Multiple linear regression analysis was used to identify the factors associated with lipid variability. Significant variables in the univariate analysis were selected as covariates for the multivariate analysis. Relationships between LDL- and HDL-cholesterol were assessed using bivariate correlations (Pearson’s correlation). Multiple comparisons among the study groups were performed with a one-way analysis of variance (ANOVA) followed by a post hoc test adjusted with a Boneferroni correction. A *p* value of less than 0.05 was considered to indicate a statistically significant difference.

## Abbreviations

CKD: chronic kidney disease
HDL: high-density lipoprotein
LDL: low-density lipoprotein
U_PCR_: urine protein-to-creatinine ratio
eGFR: estimated glomerular filtration rate
ACEIs: angiotensin converting enzyme inhibitors
ARBs: angiotensin II receptor blockers
SD: standard deviation
DM: diabetes mellitus
